# Age and gender effects on echocardiographic and histopathological cardiac phenotypes in healthy adult mice

**DOI:** 10.1002/ame2.70183

**Published:** 2026-03-24

**Authors:** Shuang Wen, Xijia Shao, Xiulin Zhang, Ningning Zhang, Xiao Chen, Guangxin Yue, Jiangping Song

**Affiliations:** ^1^ Beijing Key Laboratory of Xenotransplantation, Fuwai Hospital, National Centre for Cardiovascular Disease Chinese Academy of Medical Sciences and Peking Union Medical College Beijing China; ^2^ Department of Cardiac Surgery, Fuwai Hospital, National Center for Cardiovascular Diseases Chinese Academy of Medical Sciences and Peking Union Medical College Beijing China; ^3^ State Key Laboratory of Cardiovascular Disease, Fuwai Hospital, National Center for Cardiovascular Diseases Chinese Academy of Medical Sciences and Peking Union Medical College Beijing China; ^4^ Shenzhen Key Laboratory of Cardiovascular Disease, Fuwai Hospital Chinese Academy of Medical Sciences Shenzhen China; ^5^ Department of Cardiac Surgery, Fuwai Yunnan Hospital Chinese Academy of Medical Sciences, Affiliated Cardiovascular Hospital of Kunming Medical University Kunming China

**Keywords:** age, C57BL/6J mice, cardiac phenotype, echocardiography, gender

## Abstract

**Background:**

Cardiac structure and function undergo progressive age‐related changes influenced by sex, yet systematic characterization of these physiological variations in preclinical models remains incomplete.

**Methods:**

Using echocardiography and histopathology, we examined age‐ and sex‐dependent cardiac phenotypes in healthy adult C57BL/6J mice (*n* = 40), stratified into eight groups by age (8, 12, 20, and 30 weeks) and sex (male/female, *n* = 5 per group). Longitudinal echocardiographic assessments quantified cardiac dimensions and function, whereas histopathological analyses (hematoxylin and eosin, wheat germ agglutinin, α‐actinin and cTnI immunofluorescence, p16^INK4a^ and ATP2B1 immunohistochemistry, and Masson's trichrome staining) evaluated myocardial architecture, fibrosis, and molecular expression characteristics.

**Results:**

Female mice exhibited significant age‐dependent left ventricular dilation, increased cardiac mass, and cardiomyocyte hypertrophy, whereas males exhibited greater structural stability. Conversely, males developed pronounced interventricular septal thickening and exacerbated myocardial fibrosis at later ages (20–30 weeks). Structural protein and molecular expression remodeling may represent the underlying mechanism.

**Conclusion:**

These findings underscore the critical role of age and sex in cardiac remodeling and establish a normative reference dataset for cardiac parameters in healthy adult mice. By defining robust baseline metrics, this study enhances experimental design in cardiovascular research, improving reproducibility and translational relevance of preclinical studies.

## Graphical abstract

This study investigated the impact of age and sex on cardiac phenotypes in healthy adult C57BL/6J mice using echocardiography and histopathological analyses. Findings revealed that female mice demonstrated more pronounced global cardiac chamber dilation during aging. Conversely, male mice developed distinct age‐related septal thickening and myofibril disorganization. These results underscore the critical roles of age and sex in shaping cardiac phenotypes and provide a comprehensive reference dataset of echocardiographic and histopathological parameters for healthy adult mice across these variables. By establishing these baseline values, this study offers a vital resource to improve the reproducibility and translational relevance of preclinical studies.

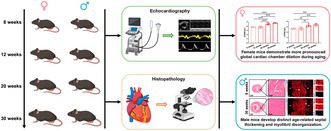

## 1 INTRODUCTION

The structure and function of the heart undergo progressive, age‐dependent physiological changes characterized by distinct morphological and functional adaptations.^1^ With advancing age, the cardiovascular system exhibits a series of intrinsic alterations, including myocardial stiffening, left ventricular hypertrophy, and reduced diastolic compliance, which collectively contribute to age‐related declines in cardiac performance.^2^, ^3^, ^4^ These age‐related modifications, although part of normal physiology, create a substrate for increased cardiovascular vulnerability in the elderly, highlighting the importance of distinguishing between adaptive cardiac aging and pathological remodeling.^5^, ^6^ In addition to aging, sex constitutes another pivotal determinant of cardiac phenotypes.^7^, ^8^ From puberty onward, differential secretion of sex hormones (particularly estrogen and testosterone) drives distinct sex‐specific cardiac remodeling patterns.^9^ At the pathophysiological level, in many studies, gender has been identified as a significant variable affecting disease risk, disease prognosis, and the effectiveness of therapeutic plans.^10^, ^11^, ^12^ For example, for patients with heart failure, the risk of all‐cause mortality in women was 0.83 that of men, and the risk of cardiovascular mortality was 0.84 that of men, showing a better prognosis.^11^ Even in the normal physiological aging process, changes in the cardiovascular system exhibit gender‐dependent differences.^13^, ^14^

As one of the most commonly used experimental animal models, C57/BL6 mouse models are widely used in research on the pathogenesis and therapeutic interventions of cardiovascular diseases.^15^ Currently, mouse models have been successfully established to simulate various human cardiovascular diseases, including cardiomyopathy, myocarditis, coronary artery disease, and Kawasaki disease.^16^, ^17^, ^18^, ^19^ However, due to the short life cycle of mice and the rapid transition of growth and development stages, the impact of age on cardiac structure and function is particularly prominent.^20^ Compared with 8‐week‐old mice, 24‐month‐old mice exhibited significant enlargement of cardiac chambers and decreased systolic function.^21^ At the same time, the regulatory role of sex hormones plays a key role in the development and maintenance of mouse heart function.^22^ Studies have shown that estrogen compound stimulation can cause cardiomyocyte (CM) apoptosis in male hypertrophic cardiomyopathy (HCM) mice but has no such effect on females.^23^ When using mice as a preclinical animal model, the selection of mice of different ages and genders can lead to different experimental results due to their physiological differences. Many previous studies have shown that the same intervention can lead to different outcomes in mice of different ages and sexes.^24^, ^25^ For instance, in a study on the protective effects of microRNA suppression on mouse heart, inhibition of the respective microRNA conferred greater protection in female mice.^25^ Therefore, in cardiovascular research using mice as animal models, it is particularly important to precisely match the age and gender parameters of the mice to minimize the impact of these confounding factors.^26^, ^27^

In this study, we performed echocardiographic examinations and cardiac histopathological staining on healthy mice grouped by different ages from 8 to 30 weeks and different genders. Subsequently, we systematically compared the differences in echocardiographic parameters and histopathological manifestations. Based on these results, we summarized the cardiac changes during the natural aging process of mice and the gender differences, and provided reference ranges for cardiac ultrasound parameters for mice of different ages and genders.

## 2 MATERIALS AND METHODS

### 2.1 Experimental mice

Forty normal adult WT C57BL/6J mice were divided into eight groups according to age and sex: 8‐week‐old male (*n* = 5), 12‐week‐old male (*n* = 5), 20‐week‐old male (*n* = 5), 30‐month‐old male (*n* = 5), 8‐week‐old female (*n* = 5), 12‐week‐old female (*n* = 5), 20‐week‐old female (*n* = 5), and 30‐month‐old female (*n* = 5).

All mice were bred and maintained in a specific pathogen‐free facility and provided with a normal diet and drinking water. Mice were bred and maintained under standardized conditions featuring group housing (five animals per cage), a 12‐h light–dark photoperiod (8:00 a.m. lights turned on), and constant environmental parameters (21°C ± 1°C; 50% ± 5% humidity). This study was approved by the Animal Ethics Committee of the Fuwai Hospital (0109‐7‐200‐ZX(X)‐2). The guidelines of Directive 2010/63/EU of the European Parliament on the protection of animals used for scientific purposes were followed in this animal experiment.

### 2.2 Two‐dimensional imaging electrocardiogram measurements

All mice in the study were acclimated for 1 week. Prior to ultrasound examination, chest hair was removed using depilatory cream. Anesthesia was induced by placing the mice in an induction chamber (isoflurane concentration: 4%–5%, gas flow rate: 1 L/min). After approximately 1 min of anesthesia induction, the mice were positioned supine on a dedicated ultrasound mouse platform maintained at a constant temperature of 37°C. The snout was secured within a breathing mask connected to the anesthesia machine. Mouse limbs were affixed to the platform's electrode pads using medical tape, and conductive paste was applied to the limbs. Coupling gel was applied to the chest to ensure optimal probe–skin contact and prevent air interference with image acquisition. Cardiac imaging was performed using a VisualSonics Vevo 3100 ultrasound system (VisualSonics, Inc.) equipped with a high‐frequency MX400 transducer (20–46 MHz). Parasternal long‐axis (PLAX) and short‐axis (PSAX) echocardiograms were acquired in B‐mode, followed by M‐mode imaging to capture cardiac motion.

Probe positioning for the PSAX view: from the PLAX view, the transducer was rotated 90° clockwise and the *y*‐axis was fine‐tuned to obtain the PSAX view. Parameters measured via M‐mode imaging at the PSAX papillary muscle level included left ventricular end‐systolic and end‐diastolic internal diameters (LVID;s, LVID;d); left ventricular posterior wall thickness at end‐systole and end‐diastole (LVPW;s, LVPW;d); stroke volume (SV = LVEDV – LVESV); and cardiac output (CO = SV × HR), where LVEDV and LVESV represent left ventricular end‐diastolic and end‐systolic volumes, and HR denotes heart rate; left ventricular ejection fraction (LVEF = SV/EDV); left ventricular fractional shortening (LVFS = ([LVID;d] – [LVID;s])/[LVID;d]); and left ventricular mass (LV mass), calculated using the area–length method^28^ as follows: LV mass (g) = 1.053 × ([LVID;d + IVS;d + LVPW;d]^3^ – [LVID;d]^3^); corrected left ventricular mass (LV mass corr = LV mass × reference weight/actual weight), where the reference weight was 20 g. Probe positioning for the PLAX: the transducer was positioned vertically, with the notch oriented toward the animal's head and rotated counterclockwise ~35°. End‐diastolic left atrial diameter (LAD;d), end‐diastolic left atrial area (LAA;d), and aortic outflow tract diameter (left ventricular outflow tract [LVOT]) were acquired using B‐mode imaging on the PLAX interface. Interventricular septum thickness at end‐systole (IVS;s) was obtained using M‐mode imaging on the PLAX interface.

### 2.3 Doppler and tissue Doppler imaging electrocardiogram measurements

The apical four‐chamber view was obtained for pulsed‐wave Doppler and tissue Doppler measurements. The mouse was positioned supine on the animal platform, which was first tilted 10°–15° to the left and then 10°–15° backward. The transducer was placed above the cardiac apex, with the imaging plane oriented at 45° to the coronal plane and the central axis of the ultrasound beam directed toward the skull, posteriorly and leftward to acquire the apical four‐chamber view. B‐mode imaging was performed to measure systolic right ventricular free wall thickness (RVFW;s) and end‐diasolic right ventricular diameter internal dimension at end‐diastole (RVD;d).

Color Doppler imaging was used to identify the location of peak left ventricular diastolic flow velocity, and the pulsed‐wave Doppler sample volume was positioned at this site of maximum flow velocity. Pulsed‐wave Doppler was employed to measure hemodynamic parameters related to left ventricular diastolic function. Within the pulsed‐wave Doppler spectrum, the characteristic biphasic mitral inflow pattern was visualized. The initial peak corresponded to passive ventricular filling during early diastole (mitral valve [MV] E), whereas the subsequent peak represented active atrial contraction filling (MV A). Simultaneously, the isovolumetric relaxation time, isovolumetric contraction time, and aortic ejection time were measured from the pulsed‐wave Doppler spectral tracing. Tissue Doppler imaging was utilized to assess mitral annular myocardial velocity. The sample volume was placed at the septal mitral annular position, and the acquired spectral tracing was used to measure the parameters MV E′ and MV A′. Using these parameters, MV E/E′, MV E/A, MV E′/A′, and left ventricular myocardial performance index (LV MPI) were calculated.

### 2.4 Histopathology

After the animals were killed and tissues were collected, cardiac specimens were fixed in 4% paraformaldehyde for 24 h and then stored in 70% ethanol. Biopsied heart tissues were processed through cassette‐based dehydration in an ascending ethanol series, paraffin embedded, and sectioned (4 μm thick). Subsequently, the sections were subjected to hematoxylin and eosin (H&E) histochemical staining.

### 2.5 Immunofluorescence and immunohistochemistry staining

Immunofluorescence (IF) staining was performed on murine cardiac cryosections in sequential incubation steps. Tissue sections were first exposed overnight to primary antibodies at 4°C and then incubated for 1 h at room temperature in darkness with fluorophore‐conjugated secondary antibodies (Zhongshan, ZF‐0311). After having been washed thrice in Phosphate‐Buffered Saline with Tween 20 (PBST), the specimens were mounted using DAPI‐embedded fluorescent medium for nuclear counterstaining. Antibody reagents included anti‐α‐actinin (Cell Signaling Technology, 69758S), wheat germ agglutinin (ThermoFisher, W32466), Cardiac Troponin I Polyclonal antibody (Proteintech, 21652‐1‐AP), Goat anti‐Mouse IgG (H+L) Cross‐Adsorbed Secondary Antibody (ThermoFisher, A‐11005), and Goat Anti‐Rabbit Recombinant Secondary Antibody (Proteintech, RGAR003). Digital imaging was subsequently obtained using the Pannoramic 250 FLASH II fluorescence slide scanner (3DHISTECH) with multispectral capture capabilities.

Immunohistochemistry staining was performed on formalin‐fixed, paraffin‐embedded human tissue sections using a standard indirect immunoperoxidase protocol. After deparaffinization in xylene and rehydration through graded ethanol, antigen retrieval was conducted by heating sections in citrate buffer (pH 6.0) at 95–100°C for 20 min. Endogenous peroxidase activity was quenched with 3% hydrogen peroxide in methanol for 15 min, and nonspecific binding sites were blocked with 5% normal goat serum in PBST for 1 h at room temperature. Sections were then incubated overnight at 4°C with primary antibodies diluted in blocking buffer: p16^INK4a^ (Proteintech, 10883‐1‐AP) and ATP2B1 (Proteintech, 30035‐1‐AP). After having been washed thrice in PBST, the slides were incubated with horseradish peroxidase–conjugated goat anti‐rabbit IgG secondary antibody for 1 h at room temperature. The immunoreaction was visualized using 3,3′‐diaminobenzidine chromogen substrate for 2–5 min, and sections were counterstained with hematoxylin. Finally, the slides were dehydrated through graded ethanol, cleared in xylene, and mounted with neutral balsam. Digital images were captured using a Pannoramic SCAN II brightfield slide scanner (3DHISTECH) with a 20× objective.

### 2.6 Masson's trichrome staining and quantitation of fibrosis

Paraffin embedding and sectioning adhered to previous protocols. Myocardial tissue sections were stained with Masson's trichrome under conditions specified by the manufacturer (Sigma‐Aldrich). Whole‐slide imaging was performed using an Axio Scan.Z1 slide scanner. Fibrotic regions (identified by blue collagen deposition) were quantified using ImageJ software (IBM) by investigators blinded to the experimental groups.

### 2.7 Statistical analysis

All values in this study are represented as mean ± standard error of the mean. Image processing, including channel splitting and counting five types of neurons, was performed using ImageJ, version 1.54f (IBM). Power calculations were performed using GraphPad Prism, version 9.5.0 (GraphPad, San Diego, CA, USA).

The data were tested for normality using Kolmogorov–Smirnov test or Shapiro–Wilk test. Age‐based differences in pooled or stratified sex groups were determined using one‐way analysis of variance (ANOVA) followed by Tukey's post hoc analysis for comparisons among multiple groups, or using an unpaired *t*‐test for comparisons of sex‐based differences within the same age. A *p*‐value <0.05 indicated a statistically significant difference. Significance was accepted for *p < 0.05, **p < 0.01, ***p < 0.001, and ****p < 0.0001.

## 3 RESULTS

### 3.1 Left ventricular dilation is more pronounced in aging females compared to males

**FIGURE 1:**
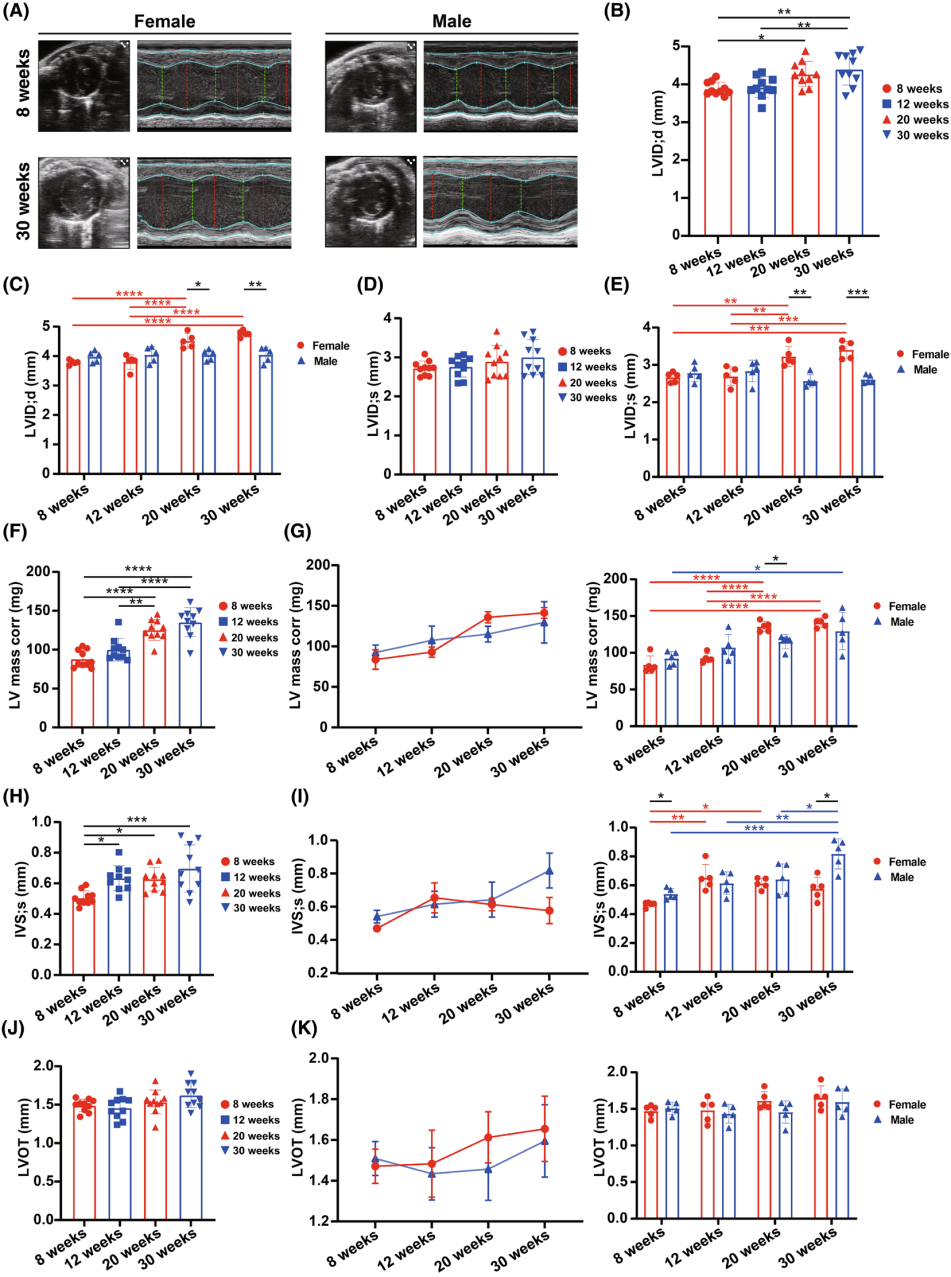
Basic parameters of cardiac structure analyzed from the echocardiographic images. (A) Representative echocardiographic images (two‐dimensional and M‐mode echocardiography in parasternal short‐axis views) of female and male mice at the age of 8 and 30 weeks. (B, C) Changes in left ventricular end‐diastolic internal diameter (LVID;d) with age in (B) pooled or (C) stratified sex groups analyzed from the echocardiographic images. (D, E) Changes in left ventricular end‐systolic internal diameter (LVID;s) with age in (D) pooled or (E) stratified sex groups analyzed from the echocardiographic images. (F, G) Changes in corrected left ventricular mass (LV mass corr) with age in (F) pooled or (G) stratified sex groups analyzed from the echocardiographic images. (H, I) Changes in interventricular septum thickness at end‐systole (IVSs) with age in (H) pooled or (I) stratified sex groups analyzed from the echocardiographic images. (J, K) Changes in left ventricular outflow tract (LVOT) diameter with age in (J) pooled or (K) stratified sex groups analyzed from the echocardiographic images. The results are presented as mean ± standard error of the mean (SEM, *n* = 5 per age‐by‐gender subgroup). Statistical analysis was performed using GraphPad Prism, version 9.5 (GraphPad, La Jolla, CA, USA). Analysis of age‐based differences in pooled (B, D, F, H, and J) or stratified (C, E, G, I, and K) sex groups was conducted using one‐way analysis of variance (ANOVA) followed by Tukey's post hoc analysis. Analysis of sex‐based differences within the same age (C, E, G, I, and K) was conducted using multiple unpaired *t*‐tests. *p < 0.05, **p < 0.01, ***p < 0.001, and ****p < 0.0001.

We conducted two‐dimensional ultrasound imaging in B‐mode and M‐mode at the PSAX to assess left ventricle (LV) structure in mice across different ages and genders (Figure 1A; Table 1). Measurements included LVID;d and LVID;s (Figure 1B–E). In the pooled sex group, LVID;d increased significantly with age, with significant differences in 20‐ and 30‐week mice compared to 8‐week mice. When stratified by gender, female mice at 20 and 30 weeks exhibited significant increases in both LVID;d and LVID;s compared to 8‐ and 12‐week counterparts, whereas male mice exhibited no significant changes. Additionally, at 20 and 30 weeks, female mice had significantly higher LVID;d and LVID;s than male mice. Corrected left ventricular mass (LV mass corr) increased significantly with age in the pooled sex group (Figure 1F). This upward trend was observed in both genders, but it was statistically significant only in female mice, with male mice exhibiting a significant difference only between 8 and 30 weeks (Figure 1G). Interventricular septum thickness at end‐systole (IVS;s) increased significantly with age in the pooled group (Figure 1H). In gender‐stratified analyses, male mice exhibited a consistent increase in IVS;s, whereas female mice exhibited irregular changes (Figure 1I). At 8 and 30 weeks, IVS;s was significantly higher in male mice than in female mice. No significant age‐related differences were observed in LVOT diameter in either pooled or gender‐stratified groups (Figure 1J,K), though female mice exhibited an age‐dependent increasing trend in LVOT (Figure 1K). These findings indicate that LV enlargement occurs with age in mice, with more pronounced changes in female mice.

**TABLE 1:** Basic parameters of cardiac structure analyzed from echocardiographic images.

	Female				Male			
	8 weeks	12 weeks	20 weeks	30 weeks	8 weeks	12 weeks	20 weeks	30 weeks
Sample size	5	5	5	5	5	5	5	5
LVID;d (mm)	3.79 ± 0.081	3.804 ± 0.252	4.519 ± 0.245	4.764 ± 0.108	3.983 ± 0.191	4.05 ± 0.268	4.039 ± 0.184	4.048 ± 0.255
LVIS;s (mm)	2.667 ± 0.135	2.696 ± 0.251	3.229 ± 0.27	3.405 ± 0.226	2.782 ± 0.225	2.834 ± 0.289	2.57 ± 0.167	2.613 ± 0.109
LV mass corr (mg)	83.755 ± 12.017	92.863 ± 6.303	135.773 ± 6.872	141.187 ± 6.539	92.374 ± 9.252	107.491 ± 17.426	115.072 ± 9.671	129.599 ± 25.457
IVS;s (mm)	0.469 ± 0.018	0.654 ± 0.091	0.613 ± 0.037	0.577 ± 0.079	0.54 ± 0.038	0.615 ± 0.078	0.642 ± 0.105	0.818 ± 0.105
LVOT (mm)	1.471 ± 0.084	1.483 ± 0.164	1.612 ± 0.126	1.655 ± 0.16	1.509 ± 0.083	1.434 ± 0.128	1.457 ± 0.154	1.595 ± 0.178

Abbreviations: IVS;s, interventricular septum thickness at end‐systole; LVID;d, left ventricular end‐diastolic internal diameter; LVID;s, left ventricular end‐systolic internal diameter; LV mass corr, corrected left ventricular mass; LVOT, left ventricular outflow tract.

### 3.2 Female mice exhibit exacerbated age‐related deterioration in left ventricular systolic function

**FIGURE 2:**
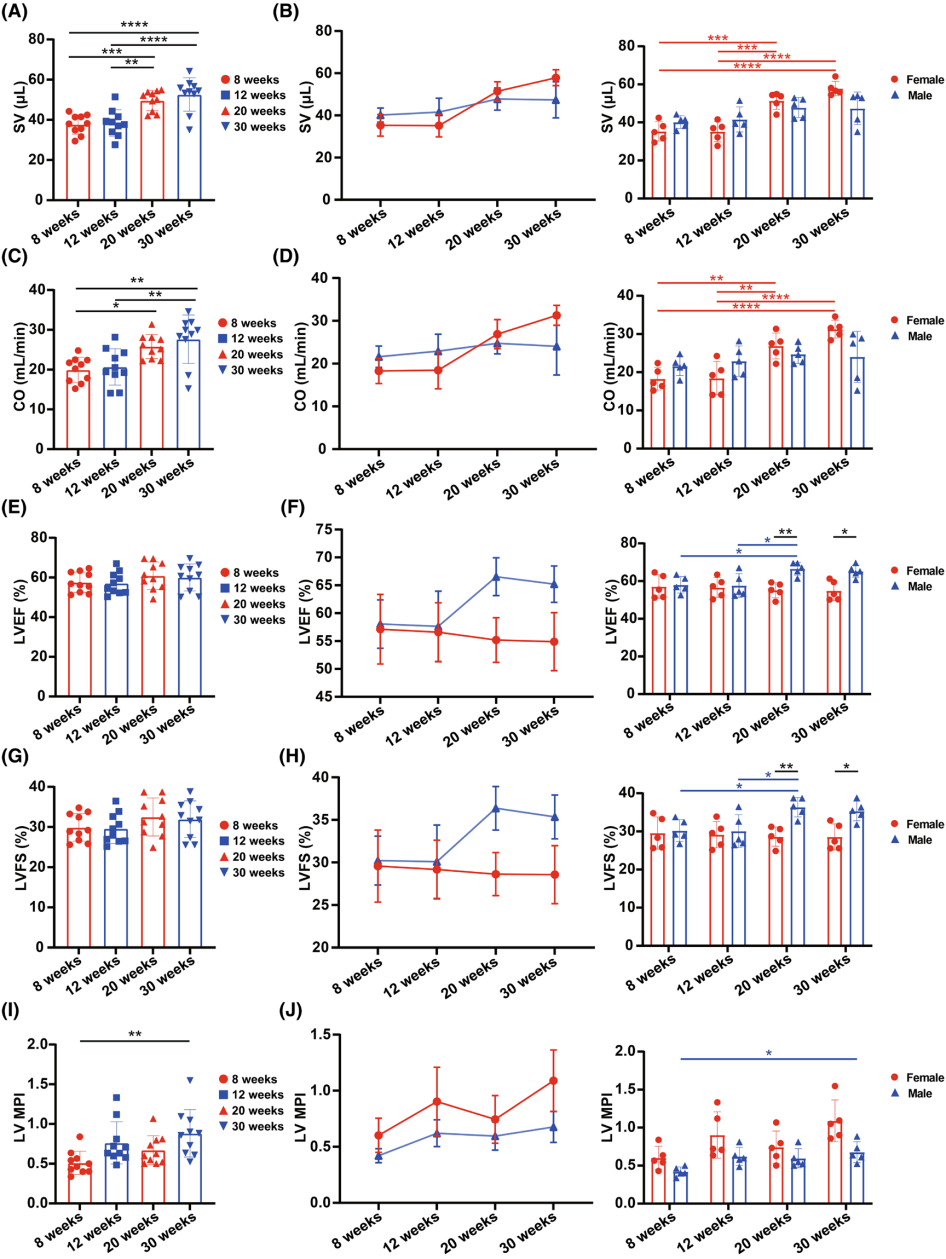
Parameters of left ventricular systolic function analyzed from the echocardiographic images. (A, B) Changes in stroke volume (SV) with age in (A) pooled or (B) stratified sex groups analyzed from the echocardiographic images. (C, D) Changes in cardiac output (CO) with age in (C) pooled or (D) stratified sex groups analyzed from the echocardiographic images. (E, F) Changes in left ventricular ejection fraction (LVEF) with age in (E) pooled or (F) stratified sex groups analyzed from the echocardiographic images. (G, H) Changes in left ventricular fractional shortening (LVFS) with age in (G) pooled or (H) stratified sex groups analyzed from the echocardiographic images. (I, J) Changes in left ventricular myocardial performance index (LV MPI) with age in (I) pooled or (J) stratified sex groups analyzed from the echocardiographic images. The results are presented as mean ± standard error of the mean (SEM, *n* = 5 per age‐by‐gender subgroup). Statistical analysis was performed using GraphPad Prism, version 9.5 (GraphPad, La Jolla, CA, USA). Analysis of age‐based differences in pooled (A, C, E, G, and I) or stratified (B, D, F, H, and J) sex groups was conducted using one‐way analysis of variance (ANOVA) followed by Tukey's post hoc analysis. Analysis of sex‐based differences within the same age (B, D, F, H, and J) was conducted using multiple unpaired *t*‐tests. *p < 0.05, **p < 0.01, ***p < 0.001, and ****p < 0.0001.

We subsequently evaluated ultrasound parameters to assess LV systolic function. SV and CO exhibited similar age‐related trends (Figure 2A–D). In pooled sex groups, SV and CO significantly increased at 20 and 30 weeks compared to 8 and 12 weeks (Figure 2A,C). In gender‐stratified analyses, both SV and CO increased with age, with female mice exhibiting more pronounced increases, evidenced by significant differences between 8 and 20 weeks, 12 and 20 weeks, 8 and 30 weeks, and 12 and 30 weeks. In male mice, an increasing trend was observed but lacked statistical significance. LVEF and LVFS, key indicators of LV systolic function, were calculated from ultrasound data (Figure 2E–H). LVEF reflects LV contractile efficiency, whereas LVFS assesses myocardial contractility.^29^ In pooled sex groups, LVEF and LVFS exhibited no significant differences across ages (Figure 2E,G). However, gender‐specific trends emerged (Figure 2F,H). In female mice, LVEF and LVFS declined with age, though not significantly. In male mice, both parameters significantly increased from 8 and 12 to 20 weeks but decreased from 20 to 30 weeks. Gender differences in LVEF and LVFS were observed at 20 and 30 weeks, underscoring the importance of considering mouse age and gender in cardiac function studies. LV MPI, measured in the four‐chamber view, increased with age in the pooled sex group (Figure 2I). Both genders exhibited an increasing trend in LV MPI, with significant differences in male mice between 8 and 30 weeks (Figure 2J). The results of these parameters are summarized in Table 2. These findings highlight age‐ and gender‐specific variations in ultrasound parameters of LV systolic function in mice, with distinct trends between sexes.

**TABLE 2:** Parameters of left ventricular systolic function analyzed from echocardiographic images.

	Female				Male			
	8 weeks	12 weeks	20 weeks	30 weeks	8 weeks	12 weeks	20 weeks	30 weeks
Sample size	5	5	5	5	5	5	5	5
SV (μL)	35.285 ± 5.14	35.198 ± 5.366	51.456 ± 4.527	57.849 ± 3.781	40.173 ± 3.336	41.581 ± 6.59	47.756 ± 5.25	47.398 ± 8.595
CO (mL/min)	18.28 ± 2.94	18.457 ± 4.357	26.877 ± 3.403	31.276 ± 2.328	21.621 ± 2.501	22.92 ± 3.959	24.737 ± 2.46	24.044 ± 6.687
LVEF (%)	57.121 ± 6.243	56.583 ± 5.286	55.193 ± 3.995	54.876 ± 5.199	58.051 ± 4.341	57.641 ± 6.294	66.547 ± 3.394	65.201 ± 3.253
LVFS (%)	29.57 ± 4.234	29.17 ± 3.446	28.632 ± 2.532	28.573 ± 3.407	30.222 ± 2.863	30.088 ± 4.306	36.368 ± 2.565	35.353 ± 2.576
LV MPI	0.602 ± 0.152	0.903 ± 0.306	0.744 ± 0.213	1.089 ± 0.274	0.421 ± 0.063	0.621 ± 0.12	0.596 ± 0.126	0.677 ± 0.137

Abbreviations: CO, cardiac output; LVEF, left ventricular ejection fraction; LVFS, left ventricular fractional shortening; LV MPI, left ventricular myocardial performance index; SV, stroke volume.

### 3.3 Female mice exhibit pronounced age‐related decline in LV active relaxation

**FIGURE 3:**
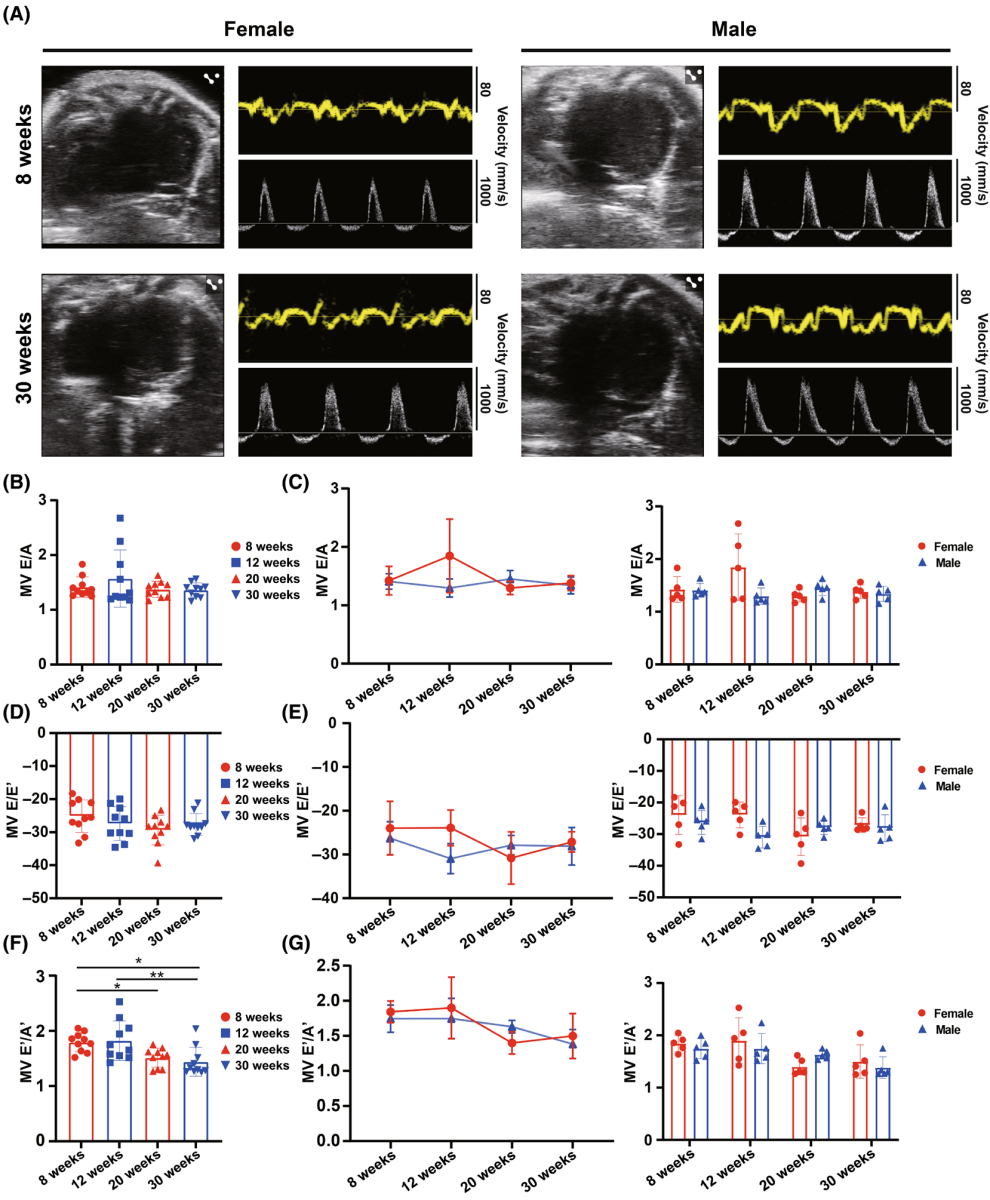
Parameters of left ventricular diastolic function analyzed from the echocardiographic Images. (A) Representative echocardiographic images (multimodal imaging in apical four‐chamber view, including two‐dimensional echocardiography, tissue Doppler imaging, and pulsed‐wave Doppler) of female and male mice at the age of 8 and 30 weeks. (B, C) Changes in mitral valve early‐to‐late diastolic flow velocity ratio (MV E/A) with age in (B) pooled or (C) stratified sex groups analyzed from the echocardiographic images. (D, E) Changes in MV E‐wave to tissue Doppler e' ratio (MV E/E′) with age in (D) pooled or (E) stratified sex groups analyzed from the echocardiographic images. (F, G) Changes in late‐to‐early diastolic mitral annular velocity ratio (A′/E′) with age in (F) pooled or (G) stratified sex groups analyzed from the echocardiographic images. The results are presented as mean ± standard error of the mean (SEM, *n* = 5 per age‐by‐gender subgroup). Statistical analysis was performed using GraphPad Prism, version 9.5 (GraphPad, La Jolla, CA, USA). Analysis of age‐based differences in pooled (B, D, and F) or stratified (C, E, and G) sex groups was conducted using one‐way analysis of variance (ANOVA) followed by Tukey's post hoc analysis. Analysis of sex‐based differences within the same age (C, E, G, and I) was conducted using multiple unpaired *t*‐tests. *p < 0.05, and **p < 0.01.

We next assessed LV diastolic function in the four‐chamber view using tissue Doppler and color Doppler to derive MV E/A, E/E′, and E′/A′ (Figure 3A; Table 3). MV E/A is a key indicator of LV filling efficiency, MV E/E′ reflects LV filling pressure, and MV E′/A′ directly indicates LV active relaxation capacity.^29^ In pooled sex groups, MV E/A exhibited no age‐related changes (Figure 3B). In gender‐stratified analyses, both male and female mice exhibited fluctuations in MV E/A within a specific range across ages, with no significant differences within or between genders at the same age (Figure 3C). Particularly, the fluctuation patterns in female and male mice were contrary at each time point. Similarly, MV E/E′ in pooled sex groups exhibited no significant age‐related changes (Figure 3D). When stratified by gender, both female and male mice exhibited fluctuations in MV E/E′, with an overall downward trend (Figure 3E). Particularly, for both MV E/A and E/E′, the fluctuation patterns in female and male mice were contrary at each time point. For MV E′/A′, a declining trend was observed in pooled sex groups, with significant reductions between 8 and 20 weeks and between 8 and 30 weeks (Figure 3F). In stratified sex groups, a similar declining trend was observed, though not significant (Figure 3G). Although the effects of age and gender on LV diastolic function were less pronounced than on LV systolic function, our findings reveal distinct age‐ and gender‐related differences.

**TABLE 3:** Parameters of left ventricular diastolic function analyzed from echocardiographic images.

	Female				Male			
	8 weeks	12 weeks	20 weeks	30 weeks	8 weeks	12 weeks	20 weeks	30 weeks
Sample size	5	5	5	5	5	5	5	5
MV E/A	1.423 ± 0.243	1.846 ± 0.63	1.297 ± 0.114	1.381 ± 0.128	1.408 ± 0.133	1.296 ± 0.155	1.451 ± 0.144	1.34 ± 0.144
MV E/E′	−23.974 ± 6.124	−23.916 ± 4.077	−30.823 ± 5.957	−27.14 ± 2.311	−26.295 ± 3.767	−30.944 ± 3.433	−27.873 ± 2.212	−28.122 ± 4.284
MV E′/A′	1.843 ± 0.154	1.898 ± 0.438	1.399 ± 0.158	1.498 ± 0.319	1.745 ± 0.194	1.747 ± 0.288	1.63 ± 0.09	1.383 ± 0.207

Abbreviation: MV, mitral valve.

### 3.4 Female mice demonstrate more pronounced global cardiac chamber dilation during aging

**FIGURE 4:**
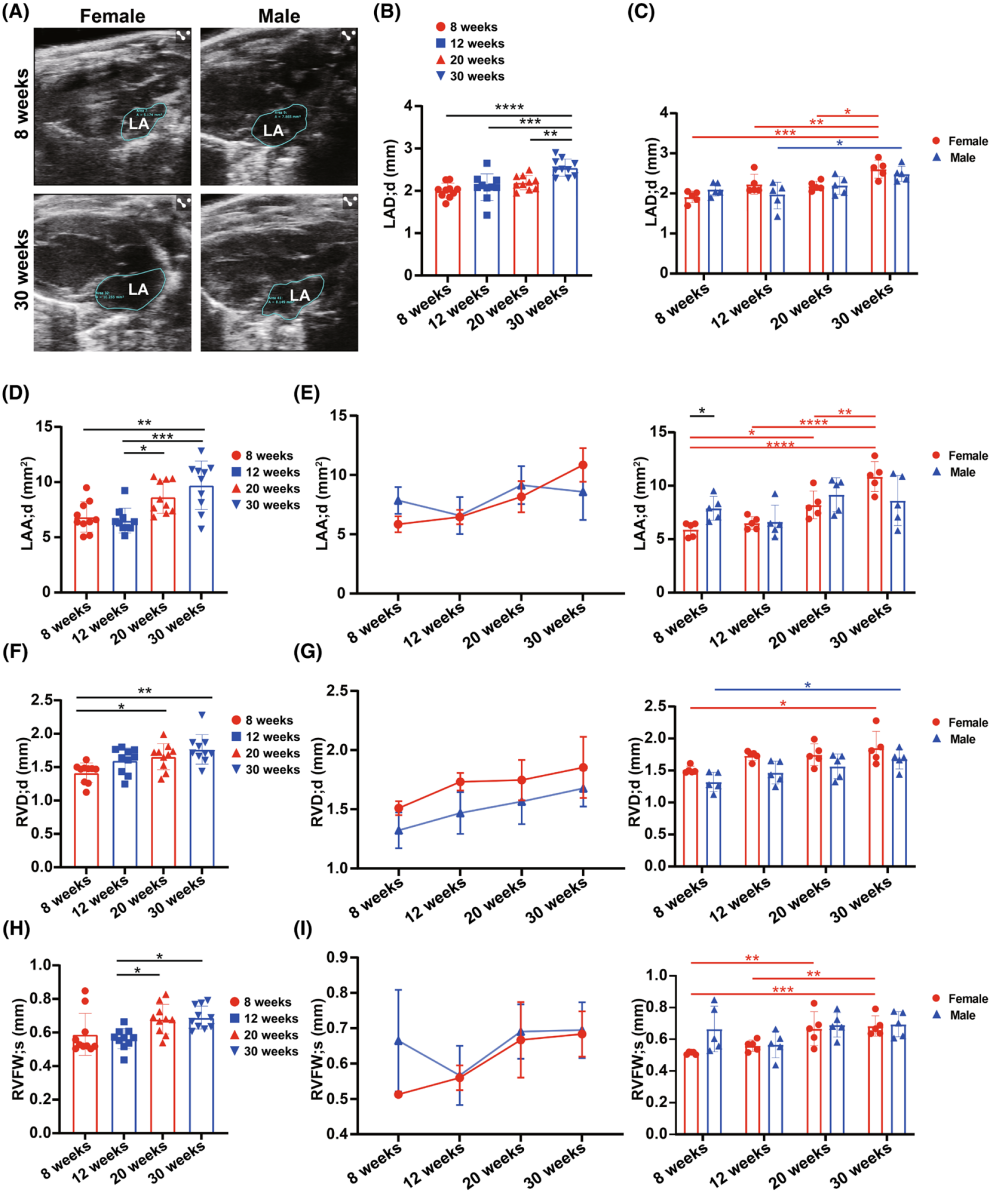
Parameters of left atrium and right ventricle analyzed from the echocardiographic images. (A) Representative echocardiographic images (two‐dimensional echocardiography in parasternal long‐axis views) of female and male mice at the age of 8 and 30 weeks. (B, C) Changes in end‐diastolic left atrial diameter (LAD;d) with age in (B) pooled or (C) stratified sex groups analyzed from the echocardiographic images. (D, E) Changes in end‐diastolic left atrial area (LAA;d) with age in (D) pooled or (E) stratified sex groups analyzed from the echocardiographic images. (F, G) Changes in end‐diastolic right ventricular diameter (RVD;d) with age in (F) pooled or (G) stratified sex groups analyzed from the echocardiographic images. (H, I) Changes in systolic right ventricular free wall thickness (RVFW;s) with age in (H) pooled or (I) stratified sex groups analyzed from the echocardiographic images. The results are presented as mean ± standard error of the mean (SEM, *n* = 5 per age‐by‐gender subgroup). Statistical analysis was performed using GraphPad Prism, version 9.5 (GraphPad, La Jolla, CA, USA). Analysis of age‐based differences in pooled (B, D, F, and H) or stratified (C, E, G, and I) sex groups was conducted using one‐way analysis of variance (ANOVA) followed by Tukey's post hoc analysis. Analysis of sex‐based differences within the same age (C, E, G, and I) was conducted using multiple unpaired *t*‐tests. *p < 0.05, **p < 0.01, ***p < 0.001, and ****p < 0.0001.

Next, we investigated ultrasound parameters of the left atrium (LA) and right ventricle (RV) to evaluate age and gender effects on these cardiac chambers (Figure 4; Table 4). LA size was assessed in the PLAX view (Figure 4A). In pooled sex groups, end‐diastolic left atrial diameter (LAD;d) significantly increased at 30 weeks (Figure 4A,B). In stratified sex groups, both female and male mice exhibited a trend of increasing LAD;d with age (Figure 4C). In female mice, LAD;d at 30 weeks was significantly larger compared to other ages, whereas in male mice, significance was observed only between 30 and 12 weeks. End‐diastolic left atrial area (LAA;d) exhibited a similar trend to LAD;d in pooled sex groups (Figure 4D). However, in stratified sex groups, a consistent and significant increase was observed only in females (Figure 4E). For RV, we measured end‐diastolic right ventricular diameter (RVD;d) and systolic RV free wall thickness (RVFW;s) (Figure 4F–I). In pooled sex groups, RVD;d exhibited a gradual increasing trend, significant between 8 and 30 weeks and between 12 and 30 weeks (Figure 4F). In stratified sex groups, RVD;d increased in both female and male mice, with significance between 8 and 30 weeks (Figure 4G). RVD;d in females was larger than in males at each age, but the difference was not significant. RVFW;s also increased with age in pooled sex groups, with significance between 12 and 20 weeks and between 12 and 30 weeks (Figure 4H). Compared to males, females exhibited a consistent and significant upward trend (Figure 4I), with comparable gender differences. These results indicate that, in addition to LV, LA and RV exhibit age‐related expansion trends with certain gender differences.

**TABLE 4:** Parameters of left atrium and right ventricle analyzed from echocardiographic images.

	Female				Male			
	8 weeks	12 weeks	20 weeks	30 weeks	8 weeks	12 weeks	20 weeks	30 weeks
Sample size	5	5	5	5	5	5	5	5
LAD;d (mm)	1.911 ± 0.143	2.23 ± 0.247	2.186 ± 0.117	2.606 ± 0.224	2.1 ± 0.161	1.947 ± 0.333	2.201 ± 0.224	2.49 ± 0.189
LAA;d (mm^2^)	5.846 ± 0.676	6.454 ± 0.606	8.17 ± 1.309	10.85 ± 1.422	7.851 ± 1.136	6.583 ± 1.561	9.142 ± 1.597	8.578 ± 2.37
RVD;d (mm)	1.509 ± 0.059	1.732 ± 0.074	1.746 ± 0.17	1.853 ± 0.258	1.322 ± 0.151	1.468 ± 0.176	1.566 ± 0.192	1.677 ± 0.154
RVFW;s (mm)	0.513 ± 0.008	0.56 ± 0.035	0.667 ± 0.107	0.684 ± 0.064	0.665 ± 0.144	0.566 ± 0.084	0.691 ± 0.077	0.695 ± 0.079

Abbreviations: LAA;d, end‐diastolic left atrial area; LAD;d, end‐diastolic left atrial diameter; RVD;d, end‐diastolic right ventricular diameter; RVFW;s, systolic right ventricular free wall thickness.

### 3.5 Interventricular septal thickening in male mice and age‐related increase in myocardial fibrosis

**FIGURE 5:**
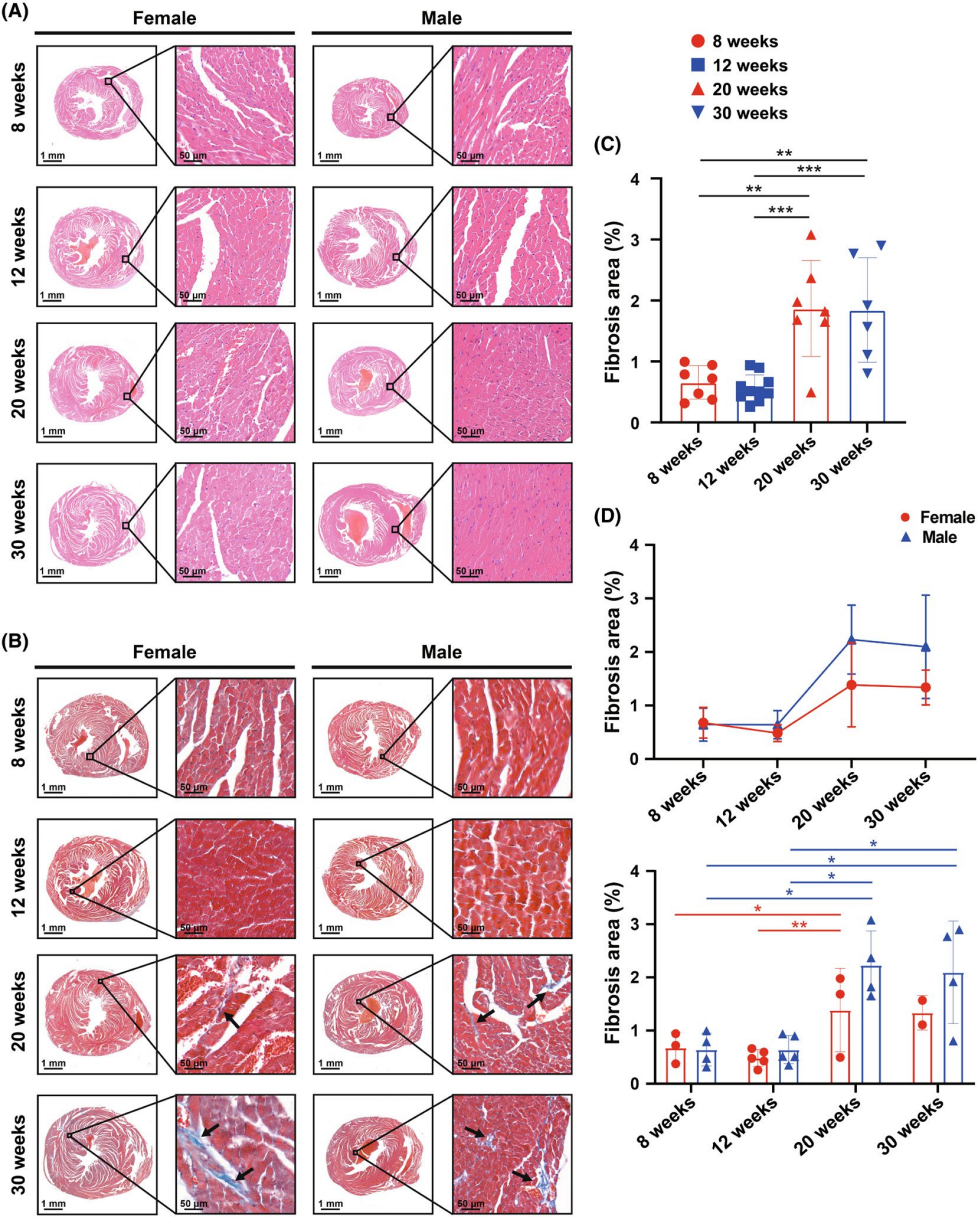
Histological alterations in myocardium revealed by histopathological staining. (A) Representative hematoxylin and eosin (H&E) staining images with magnified insets of the interventricular septum of female and male mice at the age of 8, 12, 20, and 30 weeks. (B) Representative Masson's trichrome staining images with magnified insets of female and male mice at the age of 8, 12, 20, and 30 weeks. (C, D) Changes in myocardial fibrosis area with age in (C) pooled or (D) stratified sex groups analyzed from Masson's trichrome staining. The results are presented as mean ± standard error of the mean (SEM, *n* = 2–5 per age‐by‐gender subgroup). Statistical analysis was performed using GraphPad Prism, version 9.5 (GraphPad, La Jolla, CA, USA). Analysis of age‐based differences in (C) pooled or (D) stratified sex groups was conducted using one‐way analysis of variance (ANOVA) followed by Tukey's post hoc analysis. Analysis of sex‐based differences within the same age (D) was conducted using multiple unpaired *t*‐tests. *p < 0.05, **p < 0.01, and ***p < 0.001.

**FIGURE 6:**
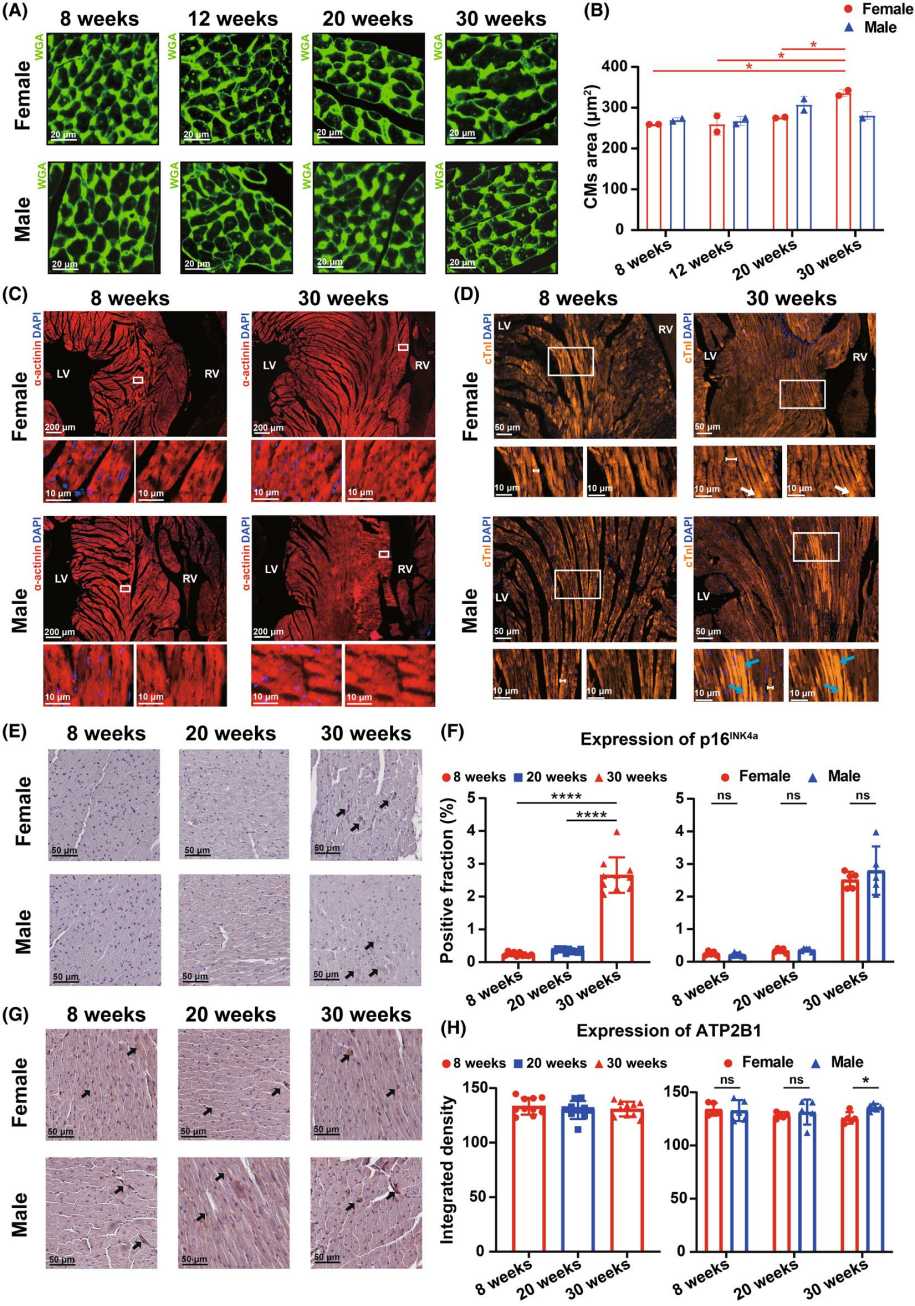
Age‐dependent and sex‐specific structural and molecular remodeling in mouse cardiomyocytes (CM). (A) Representative wheat germ agglutinin (WGA) staining images of female and male mice at the age of 8, 12, 20, and 30 weeks. (B) Changes in CM area with age in stratified sex groups analyzed from WGA staining. (C, D) Representative (C) α‐actinin and (D) cTnI immunostaining images with magnified insets of the interventricular septum of female and male mice at the age of 8 and 30 weeks. White arrow: Aggregation of cTnI. Blue arrow: Disorganized arrangement of highly compact cardiac muscle fibers. (E) Representative p16^INK4a^ immunohistochemistry (IHC) staining images. (F) Quantification of p16^INK4a^‐positive CMs. (G) Representative ATP2B1 IHC staining images. (H) Quantification of the expression level of ATP2B1 of CMs. The results are presented as mean ± standard error of the mean (SEM, *n* = 2 for WGA per age‐by‐gender subgroup, *n* = 5 for p16^INK4a^ and ATP2B1 per age‐by‐gender subgroup). Statistical analysis was performed using GraphPad Prism, version 9.5 (GraphPad, La Jolla, CA, USA). Analysis of age‐based differences in stratified sex groups was conducted using one‐way analysis of variance (ANOVA) followed by Tukey's post hoc analysis. Analysis of sex‐based differences within the same age was conducted using multiple unpaired *t*‐tests. *p < 0.05, and ****p < 0.0001.

We then employed histopathological staining to examine the histological changes in myocardial structure (Figure 5; Table 5). Hematoxylin and eosin (H&E) staining was performed to assess the basic myocardial structure (Figure 5A). Across all genders and ages, no obvious damage or deformities were observed in the cardiac chambers or ventricular walls. In male mice, IVS myocardium progressively thickened with age, whereas this change was less pronounced in female mice. No significant difference in the thickness of IVS was observed between male and female mice at 8, 12, or 20 weeks. However, by 30 weeks, the IVS thickness of male mice was significantly greater than that of female mice, consistent with ultrasound findings (Figure 1H,I). We subsequently performed Masson's trichrome staining to assess myocardial fibrosis across groups (Figure 5B–D; Table 6). With increasing age, both male and female mice exhibited increased myocardial fibrosis replacement (Figure 5B). This increase was significant in both pooled and stratified sex groups (Figure 5C,D). Additionally, the increase in myocardial fibrosis appeared to be more pronounced in male mice, though not significant (Figure 6D). These findings suggest that age and gender influences on myocardial fibrosis must be considered when using mice as models for fibrosis studies.

**TABLE 5:** CM area analyzed from WGA staining.

	8 weeks	12 weeks	20 weeks	30 weeks
*Female*				
Sample size	2	2	2	2
CM area (μm^2^)	259.401 ± 10.888	260.228 ± 35.738	276.043 ± 29.894	336.202 ± 32.028
*Male*				
Sample size	2	2	2	2
CM area (μm^2^)	270.444 ± 24.491	267.847 ± 37.501	307.79 ± 23.871	281.205 ± 29.411

Abbreviations: CM, cardiomyocyte; WGA, wheat germ agglutinin.

**TABLE 6:** Myocardial fibrosis analyzed from Masson's trichrome staining.

	8 weeks	12 weeks	20 weeks	30 weeks
*Female*				
Sample size	3	5	3	2
Fibrosis area (%)	0.679 ± 0.615	0.484 ± 0.353	1.386 ± 1.005	1.337 ± 0.832
*Male*				
Sample size	4	5	4	4
Fibrosis area (%)	0.643 ± 0.479	0.642 ± 0.548	2.23 ± 1.108	2.097 ± 1.515

### 3.6 Structural protein and molecular expression remodeling underlies age‐ and sex‐related differences in mice cardiac phenotype

To determine the cause of the altered cardiac phenotype, we performed IF staining to investigate the underlying cellular and molecular mechanisms. Wheat germ agglutinin (WGA) staining was used to quantify the cross‐sectional area of CMs (Figure 6A,B). In female mice, CM area significantly increased with age (Figure 6A,B), whereas in male mice, CM area exhibited irregular, nonsignificant fluctuations. We then performed IF staining for α‐actinin and cTnI in CMs to examine their microstructure, focusing on the IVS region (Figure 6C,D). In 8‐week mice, both male and female, actin filaments in CMs were regularly arranged with clear sarcomere staining. However, in 30‐week mice, actin filament arrangement of CMs in some IVS regions appeared to be disordered (Figure 6C), with this phenomenon being more pronounced in male mice. These results indicate that myocardial cell arrangement and structure exhibit histopathological differences across ages and genders in mice. IF staining of cTnI revealed blurred troponin complex and abnormal aggregation of cTnI in some CMs of 30‐week female mice (Figure 6D, white arrows). Furthermore, the myofiber width was increased in 30‐week female mice, which was consistent with the WGA staining results (Figure 6A,D). In 30‐week male mice, partial overlap and disorganized arrangement of myocardial fibers were observed in the interventricular septum (Figure 6D, blue arrows), findings that align with the H&E staining results. No significant widening of myofibers was detected in male mice.

To further investigate the molecular mechanism, immunohistochemical staining was performed for the senescence‐associated protein p16^INK4a^ and the calcium‐binding protein ATP2B1. In mice aged 8–20 weeks no significant changes were observed in the proportion of p16^INK4a^‐positive cells, regardless of sex (Figure 6E,F). However, a significant increase in p16^INK4a^‐positive cells was detected in 30‐week mice. This increase was consistent across both female and male mice, with no apparent sex difference. When sexes were combined, ATP2B1 expression exhibited a slight, nonsignificant downward trend with age (Figure 6G,H). Of note, ATP2B1 exhibited a mild but nonsignificant increase in 30‐week males (Figure 6H). Furthermore, in 30‐week mice, ATP2B1 expression was significantly higher in males than in females.

Together, these findings suggest that alterations in spatial organization of structural proteins and expression levels of senescence‐associated proteins and calcium‐related proteins within CMs may underlie the age‐related and sex‐dependent changes in the cardiac phenotype observed in mice.

## 4 DISCUSSION

In cardiovascular research, mice are frequently utilized as model organisms. However, mice exhibit distinct physiological characteristics. Compared to humans, mice have an average lifespan of only 2–3 years, and their hearts undergo physiological changes more rapidly and intensely over a short period.^30^ This accelerated life cycle and reproductive capacity render mouse cardiac function particularly sensitive to variations in age and gender compared with human. For example, pregnancy induces significant physiological cardiac adaptation in both humans and mice, but key differences exist in the magnitude, nature, and underlying mechanisms of these changes. In humans, the primary adaptation is a profound volume overload, leading to an increase in CO (20%–30%), primarily achieved through an increase in SV and significant heart rate elevation (15%–25%).^31^, ^32^ This is accompanied by eccentric hypertrophy, where the LV dilates (14%) and the wall thickness increases (10%) to maintain normal systolic function.^33^ In contrast, mice develop significant concentric hypertrophy, with a disproportionate increase in left ventricular wall thickness relative to chamber diameter, and rely more heavily on a significant increase in heart rate to elevate CO, with a comparatively smaller contribution from increased SV.^34^ These differences are attributed to the shorter gestation period, higher litter size, and vastly different hemodynamic demands in mice.^35^ Consequently, although the mouse is an invaluable model for clinical study, direct extrapolation of functional data to the human clinical context requires caution due to these fundamental physiological disparities. Besides, in mice, aging is associated with reduced CO, increased left ventricular wall thickness, and diminished diastolic function, due to CM loss, fibrosis, and alterations in calcium handling.^36^, ^37^ Additionally, aged mice exhibit some degree of extracellular matrix remodeling and increased cardiac fibrosis.^38^ Gender differences also play a pivotal role in the manifestation of cardiac function in mice, with disparities in cardiac structure and function between male and female mice closely linked to their sex hormone levels.^39^ These gender differences may lead to divergent outcomes in cardiovascular disease studies using mouse models. In myocardial infarction models, female animals typically exhibit less‐severe injury and faster recovery compared to male animals.^40^, ^41^ The influence of gender on cardiac function has yielded conflicting results across numerous studies.^42^ However, these studies were predominantly conducted under specific pathological conditions. Gender‐related polymorphisms in cardiac phenotype may result secondarily from distinct response mechanisms under various pathological states, rather than only from physiological differences driven by gender.^43^, ^44^ Consequently, characterizing the physiological profiles of healthy adult mice of different genders is particularly crucial.

In this study, echocardiography and histopathological analysis were used to systematically investigate the cardiac phenotypic changes in healthy adult C57BL/6J mice of several ages (8, 12, 20, and 30 weeks) commonly used in animal experiments under different sexes. Our results further validate the effects of age and sex on the structure and function of the heart in mice.

Our study revealed that in healthy adult mice, female mice exhibit more pronounced age‐related structural and functional changes in echocardiographic parameters, whereas male mice exhibit relative stability. Specifically, we observed a significant increase in heart weight with advancing age in female mice, consistent with findings in humans.^45^ Furthermore, at 20 and 30 weeks, female mice exhibited significant increases in LVID;d and LVID;s, whereas these parameters remained unchanged in male mice. However, despite ventricular enlargement, LVEF and LVFS did not significantly decline, suggesting that cardiac systolic function in female mice was not compromised. This indicates that the age‐related cardiac enlargement is physiological in nature. Such physiological expansion is likely associated with increased cardiac workload due to pregnancy.^46^, ^47^ Studies have demonstrated that estrogen β receptors are involved in exercise‐induced physiological cardiac hypertrophy.^48^ This suggests that estrogen plays a role in mediating physiological cardiac hypertrophy. At the cellular level, WGA staining confirmed an age‐dependent increase in CM cross‐sectional area in female mice, corroborating the echocardiographic findings. IF staining of cTnI revealed that CMs in 30‐week female mice exhibited significant expression and partial aggregation of cTnI, which may account for the hypertrophy of CMs. Previous studies have demonstrated that p16^INK4a^ expression in CMs increases significantly with aging and is associated with declined cardiac function.^49^ In the present study, we observed that the proportion of p16^INK4a^‐positive CMs was already significantly elevated at 30 weeks of age, despite this time point not being conventionally considered aged. This suggests that the expression of senescence‐associated proteins may precede phenotypic aging, an observation with important implications for understanding aging mechanisms and selecting the appropriate animal model.

In echocardiography assessment, healthy adult male mice exhibited greater stability compared to female mice. Specifically, regarding cardiac structure, a significant age‐related change was observed only in the IVS;d in male mice. This stability may be attributed to the slower heart rate in male mice, which implies a lower cardiac workload, as well as more consistent hormone levels compared to female mice.^50^, ^51^ Consistent with the echocardiographic evidence of interventricular septum thickening, staining for α‐actinin and cTnI revealed disordered actin filament arrangement in the CMs of the interventricular septum in male mice. These findings indicate the specific structural remodeling associated with cardiac aging.^52^ Interestingly, in terms of cardiac function, we observed a significant increase in LVEF and LVFS in male mice between 12 and 20 weeks, and a subsequent decline. This suggests that male mice may undergo changes in left ventricular systolic function during their natural maturation process. Immunohistochemical staining revealed a slight increase in the calcium‐binding protein ATP2B1 within CMs of male mice, which reached statistical significance when compared to females at 30 weeks of age (Figure 6G,H). Consistently, echocardiographic assessment demonstrated enhanced systolic function in male mice (Figure 2E–H). These findings suggest that alterations in myocardial calcium homeostasis may be associated with changes in contractile function. Because male mice in this age range are commonly used as animal models for cardiovascular disease interventions, it is crucial to account for these physiological changes in left ventricular function and the potential mechanism during experimental design.^53^, ^54^

Masson's trichrome staining of myocardial sections revealed an age‐related increase in fibrosis levels in both male and female mice, potentially associated with an increase in matrix metalloproteinases with advancing age.^38^ Furthermore, in this study, male mice exhibited more pronounced myocardial fibrosis at 20 and 30 weeks compared to female mice. Previous studies have indicated that under certain pathological conditions, such as relaxin deficiency or acute myocardial infarction, male mice exhibit more significant fibrosis and collagen deposition than female mice.^55^, ^56^ However, the degree of fibrosis observed in this study, although elevated with age, remained at relatively low levels, with no evidence of extensive pathological fibrosis. Because this study focused on healthy, nonsenescent adult mice, the causes and consequences of the observed fibrosis elevation may differ from those associated with pathological fibrosis.

In summary, this study conducted systematic echocardiographic examinations and histopathological analyses on healthy adult C57BL/6J mice of varying ages and genders, providing robust evidence to elucidate the impact of age and gender on cardiac structure and function, thereby laying a solid foundation for further research in this field. Specifically, our findings reveal that female mice exhibit more pronounced structural and functional cardiac changes during aging, including expansion of the LV and a decline in systolic function, whereas these changes are relatively less significant in male mice. On one hand, this study reaffirms the critical roles of age and gender in shaping the cardiac phenotype in mice. On the other hand, by establishing detailed echocardiographic parameters and histological data, it provides reference ranges for cardiac phenotypes across different ages and genders in mice. These reference values are crucial for optimizing the design of animal experiments related to cardiovascular diseases, enhancing the reproducibility and comparability of experimental outcomes.

## AUTHOR CONTRIBUTIONS

**Shuang Wen:** Methodology; software. **Xijia Shao:** Formal analysis; funding acquisition; writing – original draft. **Xiulin Zhang:** Conceptualization; investigation; writing – review and editing. **Ningning Zhang:** Methodology. **Xiao Chen:** Investigation; project administration. **Guangxin Yue:** Funding acquisition; resources; visualization. **Jiangping Song:** Funding acquisition; project administration; writing – review and editing.

## FUNDING INFORMATION

This work was supported by the Central High‐level Hospital Clinical Research Funding (grant no. 2025‐GSP‐GG‐38, to Guangxin Yue), the National Natural Science Foundation for Distinguished Young Scholars of China (grant no. 82125004, to Jiangping Song), the National Key Research and Development Program of China (2023YFF0724701, to Xiao Chen) and the Innovation and Entrepreneurship Training Program for College Students of Peking Union Medical College (grant no. 2025dcxm161, to Xijia Shao).

## CONFLICT OF INTEREST STATEMENT

All authors declare no competing interests.

## ETHICS STATEMENT

This study was approved by the Animal Ethics Committee of Fuwai Hospital (0109‐7‐200‐ZX(X)‐2).

## Data Availability

The datasets generated and analyzed during the current study are available from the readers and reviewers upon reasonable request. The decision on requests will be made by the corresponding author.
